# Tuberculosis in Tibetan refugee settlements in India: a human rights perspective

**DOI:** 10.3389/fpubh.2026.1799708

**Published:** 2026-04-30

**Authors:** Nawang Yanga, Michaela Hynie, Halton Quach, Jessica Vorstermans, Amrita Daftary

**Affiliations:** 1 Faculty of Health, School of Health Policy and Management, York University, Toronto, ON, Canada; 2 Dahdaleh Institute for Global Health Research, York University, Toronto, ON, Canada; 3 Department of Psychology, York University, Toronto, ON, Canada; 4 Department of Biology, York University, Toronto, ON, Canada; 5 School of Global Health, York University, Toronto, ON, Canada; 6 Centre for the AIDS Programme of Research in South Africa, University of KwaZulu-Natal, KwaZulu-Natal, South Africa

**Keywords:** human rights and health, refugee, social determinants of health, Tibetan refugees, tuberculosis

## Abstract

The statelessness and political vulnerability of Tibetans-in-exile in India are crucial drivers of tuberculosis (TB) in this population. The violent and forced displacement of Tibetans from Tibet into India in 1959 led to overcrowded and poorly resourced settlement camps that sparked a micro-epidemic of TB among exiled Tibetans, which persists to this day. This paper focuses on the diversity and precarity in legal status among Tibetans in exile in India, human rights implications and impacts on drivers of TB particularly access to housing, health care, nutrition, and employment. The absence of a refugee framework in India, notwithstanding a broadly receptive stance toward the Tibetan people and the Tibetan Government-in-Exile, intersects with and compounds these barriers. We posit a human-rights based approach, which is needed to ensure states are accountable for safeguarding fundamental human rights that are pivotal to ending TB in Tibetans in exile, and other populations facing sociolegal and health precarities.

## Introduction

In 2024, 123.2 million people were forcibly displaced worldwide and 42.7 million (34%) were refugees (1). While many refugees come from LMICs and settle in high income countries, 73% are hosted within other, often neighboring, low- to middle-income countries (LMICs) (2). Among other health challenges, refugees have a high rate of the airborne infectious disease tuberculosis (TB), significantly surpassing those seen in host populations.

TB is an old, persistent disease. Though it is treatable and curable, the year 2022 saw over 10 million cases of people with TB, and nearly 1.5 million related deaths (3). A vast majority of TB occurs in LMICs (3). India is particularly affected, representing a quarter of the world’s burden (3). As in other settings, TB in India is deeply associated with the social determinants of health. Poverty, poor living and work conditions, and undernutrition are among the leading drivers of transmission (4). Inequities in the distribution of these social determinants, along the lines of income, gender, caste, and rural residence, leave many residents with poor access to quality health care and social protections, and at higher TB risk (5). Compared to other TB-affected populations, the risk is understood to be substantially higher among refugees, as forced displacement elevates threats to housing, food and financial security, and access to social and health services (6–8).

Nearly half of the refugees and asylum seekers living in India are of Tibetan origin (8). With an estimated incidence of 431 cases of people with TB per 100,000 persons, this group of refugees has among the highest rates of TB globally (9). The social drivers of TB among Tibetans in exile in India, however, are minimally explored. Here, we describe how geographic displacement, and the subsequent isolation of the Tibetan diaspora in India have created the conditions for an unmitigated TB epidemic. We posit that greater recognition of these social and structural determinants necessitates a human rights-based response to TB that is distinct from, albeit complementary to, a biomedical response. Centering the right to health in the TB discourse places people first and advocates for the integration of human rights approaches in health policy alongside biomedical approaches (10). It emphasizes the need for context-driven TB policy and state responsibility in fulfilling legal obligations under the right to health. Globally, TB responses are increasingly informed by human rights-based approaches, but we argue that the precarious legal status of Tibetans in exile complicates its seamless application in India (11).

To guide this analysis, we adopt a conceptual framework that links structural determinants to downstream TB outcomes. At the most upstream level, legal precarity, statelessness, and governance arrangements shape access to rights, protections, and public resources. These structural determinants influence intermediate conditions including housing, nutrition, employment, and access to health care which in turn shape TB exposure, transmission, diagnostic delays, and treatment continuity. This framework allows us to trace the pathways through which socio-legal marginalization produces elevated TB risk among Tibetans in India.

## Historical context of Tibetans in India

It is impossible to unveil the nature of TB among Tibetans in India without acknowledging the traumatic migration events that lead to their heightened risk for disease. Tibet, known as the ‘roof of the world’, is situated amidst the world’s highest peaks. Following a series of sociopolitical events, Dharamsala is today the physical, constitutional and spiritual capital for the Tibetan diaspora, including the devout practice of Buddhism. In 1950, the People’s Liberation Army of the People’s Republic of China, invaded the northeastern region of Amdo and took over Tibet (12). Given their uniquely non-violent culture, there were few instances of Tibetan resistance and revolt. On March 10, 1959, some 300,000 Tibetans gathered outside of the residence of their religious leader who also headed the Tibetan government, the 14th Dalai Lama, after rumors and fears about his possible abduction (13). The Dalai Lama quietly fled into India a week later, with approval of then Prime Minister of India, Jawaharlal Nehru (13). About 80,000 Tibetans followed across the Himalayan mountains, with a minority moving to Nepal and Bhutan (13). To preserve the Tibetan identity while displaced, the Dalai Lama was supported by the Indian state in creating the Tibetan Government-in-Exile, otherwise known as the Central Tibetan Administration (CTA), in the mountainous region of Dharamsala (14). As the birthplace of Buddhism and a country that celebrated cultural and religious freedom, India was a natural choice for refuge (15).

Over the following months, the Indian government organized transit camps in Missamari, Assam and Buxa Duar, West Bengal to receive more fleeing Tibetans (16). The camps became quickly overpopulated, accelerating the spread of infectious diseases, particularly dysentery and TB (16). Being at a substantially lower altitude than they were accustomed to, Tibetans faced challenges with acclimatization. Hardships were likely compounded by sudden immersion into a new culture and language, and loss of livelihood. The first few years saw a remarkably high mortality rate (16). To address these growing concerns, many able-bodied Tibetans supported informal railroad construction zones in other elevated areas of Himachal Pradesh and Arunachal Pradesh, guided by the Dalai Lama (17). However, conditions there were also harsh. It was then that the Dalai Lama requested Nehru to allocate settlements for Tibetan people with a specific ask for them to be housed together, and not among, the general Indian population (18). Several families were granted leased plots of land, and the first Tibetan settlement was thereby established in 1960 in Bylakuppe, Karnataka (19). Increasingly, Tibetans in India are emigrating to North America, western Europe, and Australia. Border security from China has diminished opportunities for them to freely move across the Nepalese border (20).

Today, there are about 40 Tibetan settlements across India. The community has garnered the support of the Government of India (GoI) and international donors and aid agencies, implemented development programs, and built livelihoods based on agriculture, crafts, and small industry. For many, their journey is considered as exemplary in contrast to the situation facing colonized and displaced populations elsewhere (15). Even in light of these achievements, key challenges impose risks for poor health outcomes among Tibetans, in particular a heightened burden of TB (21, 22).

## Tuberculosis burden among Tibetans in India

There is little information about the health status of Tibetans in Tibet prior to 1950. They were understood to have developed high TB rates upon displacement to India, though the data are based on a limited number of studies (23). The burden of disease came to prominence in 1996–97, when a study revealed the mean annual incidence of TB among Tibetans in India was significantly higher than rates in the general Indian population (9.7 compared to 1.9 cases of people with TB per 1,000 persons), with particularly high rates in Tibetan monasteries (17.2 per 1,000 persons) (22). In 2010, incidence among Tibetans was again confirmed to be higher than in the general Indian population (431 compared to 181 cases of people with TB per 100,000 persons) (9). An exceptionally high rate of 853 per 100,000 persons was also flagged in a 2017–18 study involving children attending 11 Tibetan schools (21).

Those attending boarding schools and residing in dormitories were found to be at heightened risk for TB (with one in every five children having TB infection) compared to children attending day schools, and over a third of those diagnosed with TB were found to have been exposed to the bacillus in their household in the preceding 2 years (21). The CTA recognizes that “unhygienic surrounding(s), refugee life, low nutritional diets, lack of awareness, stress and most of all changes in the habitation from high altitude Tibet to tropical hot weather of the Indian subcontinent” are factors that contribute to TB (24). Clinicians have described TB risks associated with poor housing and congregation boarding schools and monasteries (21). Ethnographer Audrey Prost has been among few to have elucidated experiences of TB stigma among Tibetans in India, and pointed to the unique risks that may emerge across people’s migration and resettlements (25). For the most part, however, the root drivers of the TB micro-epidemic that have been elucidated among displaced and colonized populations in other contexts such as Papua New Guinea and Canada, have not been well explored in this diaspora (6, 26, 27).

## Human rights as a social determinant of health equity

Article 12 of the International Covenant on Economic, Social and Cultural Rights (ICESCR) defines the right to health as the “right of everyone to the enjoyment of the highest attainable standard of physical and mental health” (28). General Comment 14 of the ICESCR distinguishes the right to health from the right to health care. Though the right to access to health care is of paramount importance, health is also affected by a multitude of other factors extrinsic to care and the health system (28). The Covenant states that “the right to health embraces a wide range of socio-economic factors that promote conditions in which people can lead a healthy life, and extends to the underlying determinants of health, such as food and nutrition, housing, access to safe and potable water and adequate sanitation, safe and healthy working conditions, and a healthy environment.” This positions the right to health as inextricable from the equitable distribution of social determinants, indivisible from rights related to housing, nutrition, employment, safe working conditions, and income, among others. It creates an impetus to address social determinants as violations or, conversely, protections that must be addressed or built within disease prevention and treatment responses.

Public health discourses, and increasingly in TB, are replete with arguments supporting concerted attention to end health inequities. Whereas health inequality reflects “designate(d) differences, variations, and disparity in the health achievement of individuals”, health inequity refers to differences and variations that “stem from some form of injustice (29). They are avoidable differences in health status, rooted in the maldistribution of the social determinants of health, especially normative frameworks and decisions shaping policy, law and legislation (29). Health equity cannot be achieved without addressing these social (including structural) determinants.

A human rights approach to health offers one pathway to address and achieve health equity by framing the social drivers of TB in tenants in exile as “issues and patterns [of] violations” that necessitate “legal obligations for state action” (30). Proximal social determinants such as food and housing insecurity, unemployment, and literacy, as well as upstream factors such as colonization, displacement, and marginalization may thereby be considered violations of human rights at the universal, national and subnational levels (31).

Writing from the Indian context, Kumar also argues that politics, governance, and social policies cannot be separated from medicine and health, and that the right to health cannot be considered in isolation from the rights to education, livelihood, food security, and information (32). Support for rights-based approaches to health has gained traction in the global TB community. Scholars and advocates have argued that the rights to health, freedom from discrimination, privacy and confidentiality, information, and freedom and liberty are critical for TB elimination (10). They serve to protect people affected by TB from the harms driving inequities in TB, support equitable access to scientific innovations and protection from harmful infection control and care practices that may limit the mobility, freedom, and dignity of those with TB. In the case of refugees, a rights-based approach must encompass the right to health itself, and thus the additional rights relating to their historical trauma, forced displacement, and precarious economic and legal status–which place them at heightened risk for exposure to and development of TB–alongside rights related to other drivers of TB such as employment, income, housing, nutrition, and education.

## State responsibilities for protecting health as a human right

Scholars suggest that positioning the social determinants of health as legal obligations can improve state accountability and responsibility, but this is complicated for Tibetans living in India (33). Their de facto government, the CTA, also known as the Tibetan Government-in-Exile, exists as a unique entity as a state within a state. Run by Tibetan people, the CTA is funded by volunteer contributions from the Tibetan diaspora world over, the GoI and the United States State Department through agencies such as the Bureau of Populations, Refugee and Migration (PRM) and, despite some changes in 2025, the United States Agency for International Development (USAID). Beyond Tibetans-in-exile, however, the CTA lacks political legitimacy. In the event of a conflict, for example, Indian law can overrule CTA’s authority (34).

In the face of dynamic political and legal challenges posed by the statelessness that is compounded by the constrained authority and resources of the CTA, Tibetans face sufficient barriers in accessing India’s national health services beyond the limited jurisdictions of the Department of Health (DoH) of CTA. Recognizing health as a basic human right for Tibetans in India can help overcome this inequity. In India, the right to health is recognized through Article 21 of the Indian Constitution, “Protection of Life and Personal Liberty [that] no person shall be deprived of his life or personal liberty except according to procedure established by law”, and affirmed by the Supreme Court of India (35), p. 11. For example, in the case of Parmanand Katara v. Union of India, the right to access emergency medical services was held under Article 21’s right to life (36). Similarly, in the case of Paschim Banga Khet Mazdoor Samiti v. State of West Bengal, the court held that unavailability of government health care services was a violation of Article 21 (37). Several Directive Principles of State Policy in Part IV in the Indian Constitution also promise public health protections, including protections for populations that may face inequities. Article 47, for example, outlines the state’s duty to improve public health: “The State shall regard the raising of the level of nutrition and the standard of living of its people and the improvement of public health as among its primary duties” (35), p. 23.

In linking nutrition and the standard of living to improvements in public health, the Constitution makes an explicit, albeit indirect, reference to the social determinants of health. Article 46 also obligates “the State [to] promote with special care the educational and economic interests of the weaker sections of the people” and “protect them from social injustice and all forms of exploitation”, demonstrating an acknowledgement of social and health inequalities and an opportunity to protect those who are made more vulnerable by those inequalities. A recent legal environment assessment for TB, which presents some of this data, shows that although there is no single, comprehensive legislation focussed on TB, the Indian Penal Code, Consumer Protection Act, and Protection of Human Rights Act offer some degree of protections for communities affected by TB (38).

The same is not clearly written in the Tibetan constitution that forms the basis for the functioning of the CTA including the Kashag (Cabinet), the Parliament-in-Exile and the Judiciary. (Although the CTA DoH recognizes the right to health as a fundamental right in its written health policy, the same is not clearly articulated in the Tibetan Constitution.) Rather, the Tibetan Constitution lists health as a Directive Principle alongside social welfare, education, and culture. Specifically, Article 18 of the Constitution states: “To ensure public health, there should be progress in the provision of adequate facilities of hygiene and medical treatment. Efforts should be made to provide free medical treatment to the indigent; special inoculation drives should be launched to prevent chronic ailments and the spread of contagious diseases, specialized medical attention and education on environmental protection should be carried out” and “[in] particular, efforts should be made to achieve progress in the traditional Tibetan medical practice, pharmacology, and comparative study and research on Tibetan medicine vis-a-vis modern medical sciences” (39), p. 8. The focus is therefore on medical management, service delivery and protecting traditional practices, and not addressing the critical upstream drivers of health.

The CTA and the Tibetan community in India have received a laudable degree of extensive support on various fronts from the GoI. But even India, which abides by agreements that mandate the right to health, namely, the WHO Constitution, Universal Declaration of Human Rights (UDHR), and ICESCR, faces challenges in honoring a rights-based mandate, including but not limited to refugee populations (40). Governance challenges, unequal resource and wealth distribution (India’s GINI coefficient is 0.82, nearing perfect inequality), weak public health system infrastructure, lack of universal health coverage, privatization of health (over two-thirds of Indians visit private providers as a first point of medical contact), and pervasive levels of poverty, pose obstacles (41–43). A coordinated effort between the CTA and GoI, will be needed to approach the health needs of Tibetans in India from a health and human rights perspective, and there can be an opportunity to produce tangible improvements in health for all people living within the borders of India. Kenyon et al., for example, have advocated for the advancement of a “social determinants and human rights nexus” into mainstream public policy and program planning to benefit all (30).

## Opportunities and limitations of rights-based approaches

The effort to frame TB among Tibetans in India as a human rights issue is critical and timely. The United Nations High Level Meetings (UN HLM) in 2018 and 2023, political declarations on Tuberculosis have emphasized commitments to build equity, and address stigma, and human rights, into the mainstream TB response. A key pillar of the WHO End TB strategy, that aims to reduce TB incidence by 80% and mortality by 90% by 2030, is to protect and promote human rights, ethics and equity among TB affected communities (44). Member states are tasked with ensuring their TB policies explicitly incorporate issues related to human rights, especially the right to health, which encompasses the right to health care but also rights related to the underlying determinants of health. A human rights-based approach may thus increase obligations for both the Indian state and CT, and provide mechanisms to monitor the effectiveness of TB programs for Tibetans in India.

Parallel to the first UN HLM in 2018, the GoI set a target of eliminating TB by 2025, 5 years sooner than the global target (45). The National Strategic Plan for Tuberculosis Elimination (NSP), which succeeds the longstanding Revised National TB Elimination Program, has several mentions of human rights, including addressing human rights and gender related barriers in access to TB services; ensuring human rights centered care for people affected by TB; and documenting incidents of human rights infringements against people affected by TB. The NSP also categorizes refugees as vulnerable and priority populations that, “due to their reduced access to health services and the underlying determinants of health, pose challenges for TB control” (46). This may be an entryway to legislation that holds the Indian state accountable for protecting the rights of Tibetans in India in relation to TB risk and treatment.

However, while human rights frameworks provide a powerful normative and legal foundation, they may not be sufficient on their own to achieve health equity. Human rights-based approaches emphasize state accountability, assuming that legal recognition will translate into equitable access to services. However, this may not be realized in contents where governance is fragmented or ambiguous, as in the case of Tibetans navigating both the CTA and the GoI. Similarly, even with full constitutional rights, many citizen face barriers to healthcare due to poverty, discrimination, and weak public health infrastructure.

In holding states accountable for developing policies and programs that are consistent with human rights, scholars also distinguish between what states can do, are willing to do, and cannot do (47). This speaks to the notion of “progressive realization,” which recognizes that a lack of resources and information may hinder the full and immediate realization of rights outlined within treaties, and that states can take steps to fully realize those rights over a period of time (47). The CTA, far younger than the GoI, has taken smaller steps to address the right to health for Tibetan communities. Now, 60 years after its establishment, there may be fewer hurdles to prioritize these rights for Tibetans residing in India.

Across India, several populations with full citizenship status experience TB risks shaped by structural conditions similar to those affecting Tibetans in exile. Those residing in informal urban settlements in major cities such as Delhi or Mumbai face overcrowding, poor ventilation and limited access to sanitation, all of which facilitate TB transmission (48, 49). Migrant laborers who engage in construction and brick kilns face tremendous income insecurity and disruptions in the continuity of care (5, 49). TB burden in India is often associated with the caste system and tribal identity. Those belonging to scheduled castes and scheduled tribes encounter systemic geographic and social isolation, and socioeconomic marginalization that may result in delayed diagnosis for TB despite legal standing (50).

This broader landscape demonstrates that TB outbreaks in India are driven by complex intersections that include but are not limited to the incomplete realization of human rights. For many marginalized Indian citizens, “rights” exist on paper but are unfulfilled due to social inequities. Tibetans face a more fundamental structural barrier; they are altogether excluded from the rights available to others living in India. The Tibetan micro-epidemic is therefore distinguishable from that facing the Indian populace at large. However, we recognize that while legal recognition is a foundational first step toward health equity, it is insufficient to address the social determinants of TB that persist even when legal status and eligibility for protective rights are secured.

## The right to health for Tibetans with legal precarity

Despite being non-signatory to the 1951 Convention Relating to the Status of Refugees and its 1967 Protocol, India is largely accepting of Tibetans. However, those who settle in India do encounter legal precarity. The absence of an Indian refugee framework, policy or law leaves most Tibetans without an official refugee status, and without access to the benefits and rights that refugees elsewhere might hold (51). Though the Tibetan diaspora commonly label themselves as refugees, the designation has no legal or technical standing (hence we have used the term Tibetan in exile). Instead, most Tibetans are given the legal status of “foreigner”; and many others have no documentation or status at all (15).

“Foreigners” are required to hold a registration certificate (RC) that grants access to a limited number of fundamental rights under Articles 14, 21, and 25 of the Indian Constitution, such as the right to elementary education, to lease land, and to work (52). Most other rights enshrined within the Indian Constitution, however, including the right to health, own land, or work within the GoI, are limited to citizens alone. But even Tibetans who wish to hold Indian citizenship and enjoy the expanded set of rights reserved for Indians face barriers, including a lack of knowledge and practice about the application process, loss of CTA benefits, and pushback from members of their community (53). Indeed, many Tibetans forgo applying for Indian citizenship as a symbol of loyalty to Tibet and hope of a return to their homeland (54). Another barrier is assignment of citizenship; in India, this is not governed by descent or birthright. Rather, Tibetans acquire citizenship based on the citizenship status of their parents (8).

The relationship between the CTA and GoI especially complicates the status of Tibetans in India, and their capacity to hold the bodies that govern their daily lives to account. As McConnell (2011) writes, exiled Tibetans are seen as “Tibetan citizens” by the CTA, “foreign guests” in the eyes of the Indian state, and “refugees” by the rest of the world” (55). Some have gained Indian citizenship, some have RCs, and some have no legal documentation. While the CTA serves Tibetans and includes health as a Directive Principle within its constitution, it is not recognized as a state elsewhere and therefore cannot be held politically accountable. Though the Indian state can, in principle, contravene the CTA, in practice the CTA have been able to exert a significant level of administrative authority over Tibetans (56). Tibetans in India may consider themselves refugees, but without this official designation they are unable to hold the Indian state accountable. Unless they have obtained Indian citizenship, they are essentially stateless, with minimal opportunities to seek rights and protections from India, the CTA, or internationally (57, 58).

This challenges the implementation of a human rights-based approach and bears upon the manifestation of other relevant social determinants of health, described ahead, namely access to care, employment and income, and housing and food security (22).

## Access to healthcare

Access to health care among Tibetans in India is shaped by upstream structural determinants, including legal precarity, governance arrangements, and the division of responsibility between the Central Tibetan Administration (CTA) and the Government of India (GoI). These structural factors influence the availability, accessibility, and quality of services, and in turn shape intermediate barriers such as delayed care-seeking, diagnostic gaps, and treatment interruptions. Through these pathways, constraints in health system access directly affect TB detection, transmission, and continuity of care.

The right to health care has been affirmed and interpreted by the Supreme Court of India through various rulings as mentioned previously. This right is derived from the right to life and liberty under Article 21 of the Indian Constitution. Similarly, the CTA’s DoH acknowledges the right to health in its policies, though it is not recognized as a fundamental right in the Tibetan Constitution. The precarity of the Tibetans’ legal status compounded with the under-resourced Tibetan DoH posed challenges for the availability and quality of health care services, including TB care.

The DoH of CTA has established seven hospitals, five primary health centers and 36 clinics across India and Nepal (52). Access to quality care is, however, shaped by these broader structural constraints, resulting in delayed diagnosis, fragmented care pathways, and challenges in treatment adherence. Most CTA facilities, located within remote settlement areas, are minimally resourced. Many are run by community health workers that receive insufficient training to manage the wide slate of health problems (59). Barriers are compounded by poor awareness about health risks and competing beliefs about conventional and traditional medicine (23). Moreover, when seeking care at Indian facilities, Tibetans can encounter language and cultural barriers, racism, stigma, and discrimination (23, 60). Many also face unaddressed mental health problems, which affect health care seeking practices broadly (61, 62).

The CTA’s TB Prevention and Control Program has addressed TB among Tibetans since 1979 (24). With links to India’s Central TB Division, program providers receive training from the Indian National TB Institute in Bangalore (24). In its early years, the program demonstrated strong rates of TB detection, treatment coverage and completion. However, infrastructural barriers stemming from poor financing, and increased privatization has hindered a sustained response (23). Hardest to retain in TB care have been seasonal workers, who are most at risk for poor outcomes, as discussed below (23). More recent studies among Tibetans who are affected by TB point to major diagnostic delays. Non-government organizations and research institutes have attempted to cover gaps, with some success (63). Delek Hospital in Dharmsala, for example, has been commended for its management of drug-resistant TB (64). However, the consistently higher rate of TB, as compared to the general Indian population, demonstrates that significant work remains in addressing risks among Tibetans.

## Access to employment and income

Employment and income insecurity among Tibetans are closely linked to legal precarity and restricted access to formal labor markets, shaping socioeconomic vulnerability and increasing TB risk through pathways such as undernutrition and delayed care-seeking.

Article 41 of the Constitution of India directs the state to ensure the right to work, education and public assistance in certain cases of unemployment, old age, sickness and disability (35). However, Article 41 is categorized as a Directive Principle of State Policy (DPSP), which serves as a guideline for central and state governments when creating policies and laws. In contrast with Fundamental Rights, DPSPs are not directly enforceable in a court of law. Individuals cannot challenge the state for non-compliance to these principles. For Tibetans in India, then, their right to employment and income are not guaranteed, and is compounded and complicated by their precarious legal status.

Poverty and unemployment are strongly associated with TB risk, but stable income is a major challenge facing the Tibetans living in India (65). About two-thirds of the community is of lower socioeconomic status and between 25 and 30% live at or below the poverty threshold, a situation which is believed to have worsened after the COVID-19 pandemic (65). Cohorts surveyed by the CTA and other research groups over the last 25 years show unemployment rates ranging between 70 and 77%, with youth especially affected (66). In a recent economic analysis, only 25% of Tibetans were found to earn a regular income, and a third of those working were engaged in low-paying, seasonal jobs, on farms or sweater selling (67, 68). Tibetans engaged in the sweater selling business travel afar to sell goods along roadsides, with only 3% owning physical shops (69). TB rates among the under- or unemployed are found to be three times higher than those who are stably employed, and incidence is especially high (22, 23).

While Tibetans without documentation have limited opportunities, even those who hold a registration certificate face language, stigma and related social barriers that bar them equitable access to employment opportunities, in particular high-skilled work (52). This is partly tied to their education system; while Tibetans are able to be schooled distinctly in their language, opportunities for higher education are limited (15). Only 7% of Tibetans end up working outside of settlement areas (52). The CTA is a prominent employer, but unable to meet the high labor demand. “Foreigners” are also ineligible for government jobs in India, which collectively serves as one of the largest employers in the country. This leaves Tibetans at an economic disadvantage and at high risk for TB, among other diseases linked to poverty (52).

## Access to housing

Housing conditions among Tibetans in India are shaped by upstream factors including legal status, settlement policies, and governance arrangements. These structural determinants influence patterns of overcrowding and residential segregation, which directly affect TB transmission dynamics.

Although there is no explicit wording of the right to adequate housing and shelter in the Indian constitution, it is considered an extension of the Right to Life under Article 21 as previously mentioned. The ICESCR and the UDHR both recognize the right to housing as a human right (28). The forced displacement of Tibetan refugees left them unhoused and vulnerable to a core social determinant of TB (70). Tibetans arriving in India initially lived in extremely overcrowded and unsanitary conditions, where TB could easily spread (71). For example, eight inhabitants might occupy homes in poor condition originally meant for five. While additional homes and land were allocated to new waves of Tibetans, waning governmental assistance, rurality and remoteness of the settlements, and a general shortage of homes compounded housing insecurities (71). Outbreaks of infectious diseases were unsurprisingly common (23). Research suggests that TB incidence in Tibetans who stayed within settlements for longer periods of time (over 4 months) was almost three-fold higher compared to those who stayed for fewer than 4 months in a given year (22).

Today, there is comparatively little information available about settlement density and housing, with many youths migrating out in search for education and employment. However, many Tibetan monks, approximately 16,000, live in monasteries scattered across the country (52). Studies point to the greater TB incidence among monastic Tibetans who live in close quarters; likewise among children attending boarding schools (72). Congregate settings are strongly associated with greater risk of TB, and the religious, cultural and educational norms of Tibetans in exile, also shaped by their displacement, places young people especially in positions of vulnerability (21).

## Access to food and nutrition

Nutritional vulnerability among Tibetans in India is shaped by upstream structural determinants, including displacement, limited economic opportunities, and legal constraints that affect access to stable livelihoods and social protection. These structural factors influence intermediate conditions such as food insecurity and dietary inadequacy, which are well-established drivers of TB risk. Undernutrition increases susceptibility to infection, accelerates disease progression, and is associated with poorer treatment outcomes, thereby linking structural inequities directly to TB burden.

The right to adequate nutrition is implied under Article 21 of the Indian Constitution, along with Article 47, which directs states to raise the level of nutrition for its citizens. Undernutrition and malnutrition are both associated with an increase in the risk of TB, the severity of infection, the likelihood of mortality, and poor treatment success (73). Inadequate nutrition due to migration also elevates the risk for TB, which is particularly relevant for the Tibetan population (74).

Several studies have pointed to food insecurity among Tibetans in India, reflecting the downstream effects of structural and economic vulnerabilities. A 200-household survey for example, revealed high rates of food insecurity, especially among first-generation Tibetans (65). First-generation Tibetans also underutilized food ration cards distributed by the GoI due to language barriers and unfamiliarity with the Indian diet. Insecurities among second-generation Tibetans, by contrast, were tied to unstable employment and income (65). Outside of settlements, food insecurity has also been documented in monasteries, where residents face other innate risks for TB (75).

## Conclusion

This paper argues that the TB micro-epidemic among Tibetans in India, particularly those residing within refugee settlements, is the result of traumatic and forced displacement and consequential precarities related to their legal and socioeconomic status. The distinct settlement circumstances enabled for exiled Tibetans as a result of their unique relationship with India, and which enables them to preserve much of their identity, language, culture and religion quintessential in their continued struggle for freedom, have also resulted in scarcities related to housing, food, employment, and health care access, elevating their risks for TB.

Addressing TB among Tibetans in India requires translating rights-based commitments into actionable policy interventions. Targeted TB control strategies should prioritize high-risk settings such as monasteries, boarding schools, and mobile worker populations through active case finding and improved infection control. Strengthening continuity of care for seasonal and migrant workers is critical, including mechanisms such as portable treatment records and inter-state coordination. Improved integration between the Central Tibetan Administration and Government of India health systems is needed to ensure equitable access to diagnostics and treatment, alongside inclusion in national TB surveillance systems. Social protection measures including nutrition support, housing improvements, and income stabilization are also essential to address underlying vulnerabilities. These interventions require coordinated action across the CTA, Government of India, and supporting organizations.

Today, the persisting unresolved legal status for Tibetans in India remains an important determinant of health, denying them human rights protections that could mitigate many proximate risks for TB. Attaining legal recognition may enable them to experience the full right to health and, by extension, afford them the opportunity to achieve the right to health care, adequate housing, nutrition, and fair employment, and combat other harms stemming from their forced displacement. Further research into the experiences among Tibetan communities affected by TB is needed to unveil its upstream drivers and identify acceptable approaches that would uphold their right to health and cull disparities. Overcoming the TB epidemic in the Tibetan ‘refugee’ community can serve as a template for TB responses among refugee groups, strengthening the global response and India’s effort to end TB.
